# Exploring allied health research capacity in Nigeria: a qualitative study of enablers and barriers

**DOI:** 10.1186/s12913-025-13942-9

**Published:** 2025-12-23

**Authors:** Iriagbonse Iyabo Osaigbovo, Esohe Olivia Ogboghodo, Chigozie Okwudili Obaseki, Joy Chinyere Nwaogwugwu

**Affiliations:** 1 Department of Medical Microbiology, School of Basic Clinical Sciences, College of Medical Sciences, University of Benin, Benin City, Nigeria; 2 Department of Public Health and Community Medicine, School of Medicine, College of Medical Sciences, University of Benin, Benin City, Nigeria; 3 Department of Physiotherapy, University of Benin Teaching Hospital, Benin City, Nigeria; 4 Department of Public Health and Community Medicine, University of Benin Teaching Hospital, Benin City, Nigeria

**Keywords:** Allied health, Research capacity, Qualitative study, Thematic analysis, Nigeria, Health systems

## Abstract

**Background:**

Allied health professionals are integral to patient care, yet in low- and middle-income countries, their capacity to engage in research to improve the evidence base of their services remains underdeveloped. In Nigeria, most studies on research capacity are physician-focused with only limited empirical evidence for allied health cadres. This study explored the lived experiences, motivations, institutional enablers, and systemic barriers influencing research capacity among allied health professionals in a Nigerian tertiary hospital.

**Methods:**

A qualitative descriptive study was undertaken at the University of Benin Teaching Hospital. Six focus group discussions were conducted with pharmacists, medical laboratory scientists, radiographers, physiotherapists, nurses, and paramedics, alongside four key informant interviews with departmental and unit heads. Data were transcribed verbatim and analysed using reflexive thematic analysis. Rigour was maintained by triangulating data sources, peer debriefing, reflexive journalling, and an audit trail.

**Results:**

Six themes were identified: (i) research exposure and experience, largely confined to undergraduate projects with limited methodological competence; (ii) fragmented and inequitable access to training; (iii) weak departmental and institutional infrastructure including limited funding and translation pathways (iv) intrinsic motivation driven by professional growth and patient outcomes, yet undermined by workload and time pressures; (v) systemic barriers such as ethics delays, poor interprofessional collaboration, and resource constraints; and (vi) recommendations such as protected research time, internal micro-grants, structured mentorship, departmental research units, and improved access to quality journals.

**Conclusions:**

Although AHPs demonstrated strong intrinsic motivation, structural constraints at team and organisational levels limit research engagement. Institutional reforms that embed mentorship, protected time and modest internal funding are feasible first steps to strengthen AHP research capacity at the study site. Further research should include multisite studies that explore the generalizability of the findings at country level and implementation studies that test, refine and scale interventions that strengthen allied health research capacity and, ultimately, improve patient care.

**Supplementary Information:**

The online version contains supplementary material available at 10.1186/s12913-025-13942-9.

## Background

The World Health Organization (WHO) recognises research as a core competency for all health professionals and an integral component of health systems strengthening [1]. High-quality research provides the evidence base that informs health service planning, decision making and improvements in policy and practice [2]. Accordingly, the health-related Sustainable Development Goals support the adoption of new strategies to strengthen the capacity of healthcare workers in all countries to perform their job and engage in research [3]. In low- and middle-income countries (LMIC), the capacity for locally-led, and contextually relevant health research remains grossly underdeveloped [4]. Systematic reviews point to persistent deficits in training, mentorship, funding, and institutional support for research and the need for research capacity development in the health work force is a yawning gap that requires filling [2, 4]. This gap is most acute among non-physician cadres, underscoring the need to embed research competence across all health professions.

Collectively, allied health professionals (AHPs) represent a substantial proportion of the human resources for health in most countries, providing essential diagnostic, therapeutic, and rehabilitative services that underpin effective health systems worldwide [5]. Their engagement in research is therefore vital to advancing evidence-based practice, generating contextually relevant knowledge, and improving patient outcomes [2]. Consequently, several high-income countries, notably the United Kingdom and Australia, have made significant progress in building AHP research capacity within their health systems and there has been a proliferation of empirical studies measuring research performance and competencies in this cadre of healthcare workers [6]. Conversely, AHP research capacity and assessments remain neglected in LMICs where they are especially needed [5]. In Nigeria, for example, the few existing studies on research capacity assessment and development in health professionals are predominantly physician-focused, leaving allied cadres such as nurses, pharmacists, radiographers, physiotherapists, medical laboratory scientists, and paramedics underexplored and poorly documented [7–9]. This gap limits understanding of how AHPs perceive, access, and navigate research opportunities within institutional and policy environments.

Assessing research capacity rests on a multilevel conceptual framework that encompasses individual (skills and confidence), team (collaboration and mentorship), and organisational (policies, structures, and funding) dimensions [10]. Qualitative inquiry is particularly well suited to uncovering the lived experiences of healthcare workers at these levels, offering nuanced insights into motivations, barriers, and enablers that cannot be fully captured through quantitative measures. This method of enquiry was selected to explore the lived experiences of AHPs in a Nigerian teaching hospital, focusing on their exposure to research, training opportunities, institutional and departmental supports, motivations, and perceived barriers. By capturing perspectives across multiple allied health cadres, this qualitative study provides a detailed account of the systemic and professional factors shaping allied health research engagement in a typical Nigerian teaching hospital. The findings aim to inform feasible institutional reforms that can strengthen research capacity, enhance professional development, and embed research as a routine component of allied health practice.

## Methods

### Study design

This study formed the qualitative arm of a larger mixed-methods project that examined research capacity and culture among allied health professionals at the University of Benin Teaching Hospital (UBTH). A qualitative descriptive design, situated within an interpretivist paradigm, was adopted to capture participants’ lived experiences of research engagement, institutional support, and systemic barriers [11]. 

### Study setting

The study was conducted at the UBTH, a federal tertiary hospital located in Benin City, Edo state, southern Nigeria. Established in 1973, UBTH provides specialist care to a catchment population of over ten million and serves as a training centre for undergraduate and postgraduate students across medicine, nursing, pharmacy, physiotherapy, radiography, medical laboratory science, and other allied health disciplines. Despite this mandate, anecdotal evidence suggests that allied health research capacity within the institution remains underdeveloped [12]. 

### Participants, eligibility and sampling

AHPs, for the purpose of this study, includes all persons besides physicians who provide health-related services contributing to the diagnosis, treatment, and prevention of illness and disease, as well as the promotion of health and wellness. They include nurses, pharmacists, physiotherapists, paramedics, medical laboratory scientists and radiographers.

Two categories of participants were recruited: (i) practicing AHPs from nursing, pharmacy, medical laboratory science, radiography, physiotherapy, and paramedic services, and (ii) Heads of Department (HOD) and Unit Heads from nursing, radiography, pharmacy, and medical laboratory science.

Inclusion criteria were current employment in an eligible AHP cadre at UBTH; a minimum of six months continuous service in the unit or department; and willingness to provide written informed consent. Temporary/locum staff, staff who had spent less than 6 months in service, staff on prolonged leave during data collection, and individuals involved in instrument development/piloting were excluded from participating in the study.

Maximum variation, a type of purposive sampling, was used to ensure we captured diverse perspectives across different strata namely professional cadre (six cadres), gender (male/female) and professional experience (< 5, 5–10, 11–20, > 20 years in service). Potential participants were identified from departmental staff lists and purposively invited to meet these strata. Recruitment continued until sufficient information power was achieved for the focused study aim [13]. 

### Data collection

#### Rationale for data collection method

We selected cadre-specific focus group discussions (FGDs) as the primary data-collection method to elicit shared professional norms, collective experiences and intra-cadre dynamics that are often revealed in group settings; FGDs were expected to facilitate the generation of comparative viewpoints and the articulation of common challenges within professional groups [14, 15]. Key informant interviews (KIIs) with HODs supplemented FGDs by eliciting managerial and institutional perspectives that may not surface in staff groups. This combination enabled triangulation across individual, team and organisational levels.

#### Data collection instruments

The data collection instruments, namely the FGD and KII guides, were specifically developed for the purpose of this study. Their design was informed by a targeted review of relevant literature on research capacity among allied healthcare workers, notably Matus et al., Cooke, and Sambunjak et al., as well as consultation with qualitative health research subject-matter experts [2, 10, 16]. The FGD guide explored participants’ self-assessment of their research skills, experiences with training and institutional support, and recommendations for strengthening research capacity. The KII guide was tailored to elicit insights from departmental and institutional leaders regarding systemic enablers and barriers to allied health research engagement within the study setting.

Both guides were piloted internally to ensure clarity and contextual appropriateness before being used in the main study. The full English language versions of the FGD and KII guides are available as supplementary materials (see Additional file 1 and Additional file 2) to enhance transparency and reproducibility.

#### Research team

The multidisciplinary data-collection team comprised E.O.O. (Associate Professor of Public Health, > 10 years qualitative research experience) and J. C. N. (MSc, qualitative field researcher and coding lead, 4 years’ experience) as team leads supported by mid-career allied health academics, and early-career research assistants. To mitigate interviewer variability and potential bias, facilitators completed a calibration workshop (mock FGDs, joint pilot interviews and joint coding of a pilot transcript) prior to fieldwork. Where feasible, investigators avoided facilitating FGDs that included their direct reports to reduce power dynamics. Reflexivity was also ensured through pre-collection journalling, peer debriefing, and explicit acknowledgement of E.O.O’s institutional leadership role. Disciplinary diversity enriched data interpretation, while reflexive memos and an audit trail enhanced transparency.

#### Process

Between March and June 2025, six FGDs and four KIIs were conducted. FGDs comprised 6–10 participants per group, while KIIs involved one head per department/unit. In total, 48 allied health professionals and 4 heads participated. Sample adequacy was guided by the principle of information power, with the focused study aim, cadre diversity, and richness of dialogue supporting sufficiency of the data corpus [13]. Discussions lasted 60–90 min and KIIs, 45–60 min. Facilitators emphasised the non-evaluative, confidential nature of the discussions, and offered participants the option of privately conveying their views to the facilitator to minimise social desirability bias. All sessions were conducted in English, audio-recorded with permission, and supplemented by contemporaneous field notes. FGDs were cadre-specific to minimise hierarchical influence. Data collection continued until thematic sufficiency was achieved.

### Data management and analysis

Audio recordings were transcribed verbatim by trained research assistants and checked against recordings for accuracy by the primary analyst (J.C.N.). Transcripts were anonymized and stored on encrypted institutional servers with access restricted to the research team. De-identified transcripts were imported into NVivo 12 (QSR International) for coding and analysis.

Reflexive thematic analysis followed Braun and Clarke’s six phases: familiarisation, coding, theme development, review, definition, and reporting [11]. The analytic team comprised J.C.N. (primary coder - conducted line-by-line inductive coding in NVivo), E.O.O. (senior analyst - led theme synthesis and interpretive decisions) and I.I.O. (independent co-coder - double-coded a purposive 20% sample of transcripts). J.C.N. carried out initial open coding to capture participants’ terms and meanings; codes were iteratively clustered into subthemes and broader themes through weekly analytic meetings. Discrepancies in coding were resolved through reflexive discussion and consensus; unresolved items were documented in the audit trail and revisited after additional review. This reflexive consensus process prioritised interpretive depth and transparency over reliance on inter-rater reliability statistics, consistent with contemporary practice for reflexive thematic analysis [11]. 

For transparency, we provide a worked analytic example. A single quotation - *“There is no budget for research; if you want to do a project*,* it is from your pocket”* - was initially coded as *no departmental funding* and *personal funding burden*; similar codes (for example *absence of budget lines*, *self-funding for workshops*) were grouped as the subtheme *absence of funding streams*, which contributed to the higher-order theme *institutional and departmental support*. The full coding tree (themes/subthemes/illustrative quotes) and selected coded excerpts are provided in Additional file 3.

#### Framework mapping and exploration of theme interactions

**Fig. 1:**
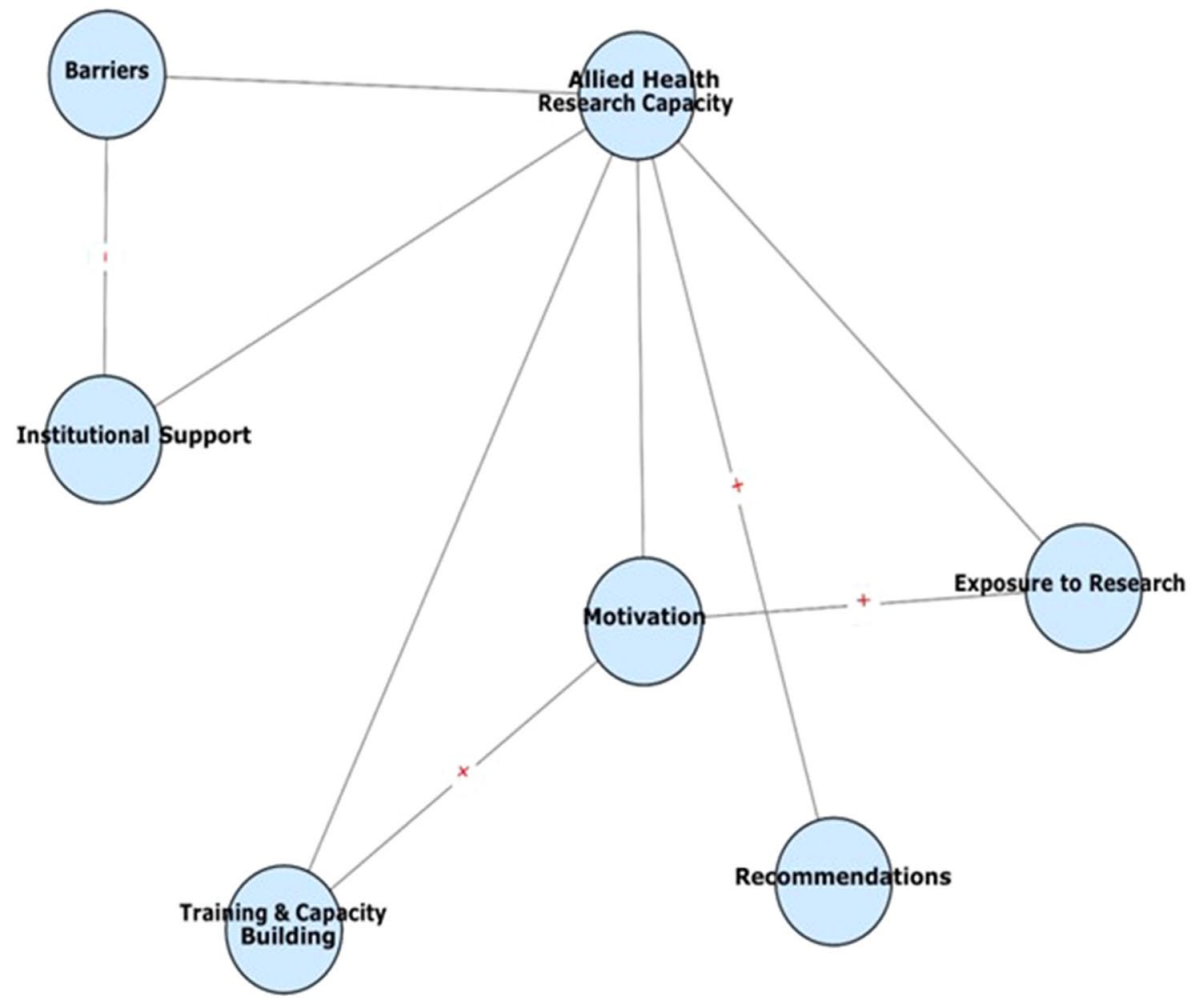
Thematic map of allied health research capacity

Our primary analytic stance was inductive; however, during interpretation we mapped emergent themes deductively onto Cooke’s research capacity building framework (individual, team, and organisational domains) to enhance theoretical coherence and policy relevance [10]. We explicitly interrogated inter-theme relationships using analytic memos and NVivo visual models, producing a thematic map (Fig. 1) that illustrates reinforcing and inhibiting pathways between individual motivation, training access, departmental support and institutional structures.

#### Determination of adequacy (information power) and stopping criteria

We assessed sample adequacy using the information power framework: the focused aim of this study, the high quality and richness of dialogues, the established relevance of participant specificity (maximum variation sampling), and the transparency of analytic strategy all supported sufficiency of the dataset [13]. Data collection ceased when two consecutive FGDs and one subsequent KII generated no new substantive codes or conceptual insights within the analytic frame.

### Trustworthiness and reflexivity strategies

We employed multiple strategies to enhance the rigor and trustworthiness of our findings, considering the pillars of reliability in qualitative research: credibility, dependability, confirmability and transferability [17]. Credibility was enhanced through method triangulation (FGDs + KIIs), investigator triangulation and double-coding as well as explicit reporting of negative/divergent cases identified during analysis. We ensured confirmability through member-checking by sharing summaries of emergent themes with a purposive subset of eight participants (one per FGD and two KII respondents) who confirmed the accuracy of thematic summaries and suggested minor wording clarifications that were incorporated into theme labels and illustrative language (for example, the subtheme ‘*workload/time pressures’* was re-worded to ‘*clinical workload and lack of protected research time’* on participants’ suggestion). We also engaged in peer debriefing and all team members practiced reflexive journalling. Dependability was achieved through maintenance of an audit trail containing original transcripts, coding frameworks across iterations, reflexive memos and decision logs. Finally, transferability of our research findings is supported by the thick description of the study context, sampling strategy and participant characteristics.

### Ethics

Ethical approval was obtained from the UBTH Health Research and Ethics Committee (Protocol No. ADM/E 22/A/VOL. VII/4831171790). All participants provided written informed consent prior to voluntary participation. The study was carried out in compliance with the Helsinki Declaration.

## Results

### Participant characteristics

A total of 48 allied health professionals participated in the FGDs, representing pharmacy, medical laboratory science, radiography, physiotherapy, nursing, and paramedic services. Participants ranged from early-career practitioners (< 5 years of service) to senior staff (> 20 years). The KIIs included two Heads of Department (nursing and pharmacy) and two heads of unit (radiography, and medical laboratory science). Gender balance was achieved across cadres. Table 1 shows the characteristics of all 52 participants. The cadre diversity and range of experience strengthened the information power of the dataset and supported cross-cadre comparison of themes. Additional detail on the coding framework and selected coded excerpts is provided in Additional file 3 (coding tree).

**Table 1:** Participant characteristics (*N* = 52)

Category	Subgroup	*n*	%
Cadre	Pharmacy	10	19.2
	Medical Laboratory Science	9	17.3
	Radiography	7	13.5
	Physiotherapy	8	15.4
	Nursing	10	19.2
	Paramedics	4	7.7
Role	Allied health professionals	48	92.3
	Heads of Department/Units	4	7.7
Gender	Male	24	46.2
	Female	28	53.8
Years of Experience	< 5 years	12	23.1
	5–10 years	15	28.8
	11–20 years	14	26.9
	> 20 years	11	21.2

### Thematic findings

Analysis yielded six interconnected themes (Fig. 1): (1) exposure to and experience with research, (2) research training and capacity building, (3) institutional and departmental support, (4) motivation for research engagement, (5) barriers to participation, and (6) recommendations for strengthening capacity. Each theme contains 1–2 subthemes that illuminate cadre-specific patterns and divergence; where relevant we indicate whether findings were raised in FGDs, KIIs, or both.

Illustrative quotations are presented to substantiate findings (Table 2).

This thematic map illustrates the six major themes identified in the qualitative analysis of allied health professionals’ (AHPs) experiences with research in the University of Benin Teaching Hospital. The central node (Allied Health Research Capacity) is surrounded by interconnected themes: exposure to research, training and capacity building, institutional support, motivation, barriers, and recommendations. Arrows indicate directional relationships: motivation was positively linked to exposure and training, while barriers exerted a negative influence on institutional support. Recommendations were viewed as pathways with the potential to strengthen overall research capacity. The map highlights the dynamic interplay between individual enthusiasm and systemic constraints, underscoring the multi-level strategies required to build sustainable AHP research cultures in resource-constrained settings.

### Theme 1: Exposure to and experience with research

Subthemes: (a) undergraduate-confined exposure; (b) limited post-qualification opportunities Most participants described limited exposure to research, typically confined to compulsory undergraduate or postgraduate projects. Beyond training years, opportunities to engage were scarce. Participants reported confidence in basic tasks such as literature review but expressed discomfort with statistical analysis and manuscript writing. These experiences were reported across cadres in FGDs and echoed by HODs in KIIs.

In school, we had to do projects, but after that, there are hardly opportunities to continue. (Radiographer, male)

I can design questionnaires, but when it comes to analysis, I depend on others. (Physiotherapist, female)

A minority of participants-typically those attached to academic units-reported ongoing research involvement post-qualification and described institutional supports that enabled continuity. This represented a negative/divergent case.

In my unit we have a weekly research seminar, and that keeps people involved. (KII, Departmental Head)

### Theme 2: Research training and capacity building

Subthemes: (a) episodic workshops without follow-up; (b) self-funded and online learning adaptations.

Training opportunities were inconsistent and uneven across cadres. Workshops and short courses were described as sporadic and usually self-funded. Some participants relied on online courses and informal mentorship to build skills.

I attended a research workshop once, but there were no follow-up trainings. (Laboratory Scientist, male)

Most times, you pay for workshops yourself, and many cannot afford that. (Nurse, female)

### Theme 3: Institutional and departmental support

Subthemes: (a) absence of funding and research units; (b) weak translation of findings into practice/policy.

Perceptions of institutional support were largely negative. Departments lacked research units, dedicated budgets, or policies to encourage staff research. Participants noted that completed studies were rarely applied to service delivery.

There is no budget for research; if you want to do a project, it is from your pocket. (Pharmacist, male)

Even when we complete research, the findings are not implemented. (Radiographer, female)

HODs recognised these gaps and, in KIIs, acknowledged the absence of formal structures to support staff research, citing competing clinical priorities and constrained institutional resources.

**Table 2:** Themes and illustrative quotations

Theme	Illustrative Quotes
1. Exposure to Research	*“In school*,* we had to do projects*,* but after that*,* there are hardly opportunities to continue.”* (Radiographer, male) *“I can design questionnaires*,* but when it comes to analysis*,* I depend on others.”* (Physiotherapist, female)
2. Training & Capacity Building	*“I attended a research workshop once*,* but there were no follow-up trainings.”* (Lab Scientist, male) *“Most times*,* you pay for workshops yourself*,* and many cannot afford that.”* (Nurse, female)
3. Institutional Support	*“There is no budget for research; if you want to do a project*,* it is from your pocket.”* (Pharmacist, male) *“Even when we complete research*,* the findings are not implemented.”* (Radiographer, female)
4. Motivation	*“I want to do research because it improves how I care for patients.”* (Nurse, female) *“Working with a mentor motivated me to write and learn more.”* (Pharmacist, female)
5. Barriers	*“Ethics approval takes too long; by the time it comes*,* the interest may be gone.”* (Pharmacist, male) *“Our workload leaves little or no time for research.”* (Paramedic, male)
6. Recommendations	*“If we had even one day a week for research*,* it would make a difference.”* (Physiotherapist, male) *“There should be a formal mentorship scheme*,* not just informal guidance.”* (Nurse, female)

### Theme 4: Motivation for research engagement

Subthemes: (a) intrinsic motivation linked to patient care and professional growth; (b) motivation strengthened by mentorship/role models.

Despite systemic challenges, participants expressed strong intrinsic motivation. They cited professional growth, career progression, and improved patient care as primary drivers. Mentorship, where available, was also described as a source of encouragement.

I want to do research because it improves how I care for patients. (Nurse, female)

Working with a mentor motivated me to write and learn more. (Pharmacist, female)

Several KIIs emphasised that visible role models and recognition of research activity were crucial for sustaining staff motivation.

### Theme 5: Barriers to participation

Subthemes: (a) structural barriers (funding, ethics delays, infrastructure); (b) workload/time constraints and poor interprofessional collaboration.

Barriers were wide-ranging and consistently reported across cadres. These included inadequate funding, prolonged ethical approval processes, poor interprofessional collaboration, lack of time due to workload, and unreliable infrastructure such as power supply.

Ethics approval takes too long; by the time it comes, the interest may be gone. (Pharmacist, male)

Our clinical workload leaves little or no time for research. (Paramedic, male)

Sometimes you cannot analyse samples because of power outages and lack of reagents. (Medical Laboratory Scientist, male)

KIIs confirmed that institutional processes (including ethics review) and infrastructure limitations were persistent institutional challenges.

### Theme 6: Recommendations for strengthening research capacity

Subthemes: (a) pragmatic institutional levers (protected time, micro-grants); (b) structural reforms (mentorship schemes, departmental research units, access to journals).

Participants offered practical strategies to improve research engagement. This included provision of protected research time, internal micro-grants, structured mentorship, establishment of departmental research units, and improved access to journals and training opportunities.

If we had even one day a week for research, it would make a difference. (Physiotherapist, male)

Small institutional grants could help, even just to buy consumables.” (Laboratory Scientist, male)

There should be a formal mentorship scheme, not just informal guidance. (Nurse, female)

### Theme interactions and visual mapping

The themes form an interconnected system: limited exposure and inconsistent training undermined individual competence, which in turn reduced outputs and weakened the organisational case for investment. Institutional deficits (funding, ethics delays, infrastructure) fed back to constrain protected time and mentorship, producing a self-reinforcing cycle of low research productivity. Figure 1 visualizes these interactions and highlights the recommended entry points (protected time, micro-grants, formal mentorship hubs) for interrupting the negative cycle.

## Discussion

This study provides a rare, in-depth exploration of AHPs lived experiences with research in a Nigerian tertiary hospital thereby contributing an LMIC narrative to the debate on AHP research capacity which has hitherto been dominated by voices from the global North. AHPs expressed strong intrinsic motivation to conduct research; ironically, they encountered systemic and institutional barriers that limited their ability to transform this enthusiasm into sustained scholarly output. Although this tension between motivation and structural constraints has been described in other LMIC contexts, our study offers qualitative depth into how these barriers are experienced, negotiated, and understood by diverse allied health cadres within a Nigerian tertiary hospital [4]. By situating our interpretation within Cooke’s research capacity-building framework, we move beyond enumerating barriers to explicating the mechanisms through which individual motivation for research is shaped, suppressed or sustained within institutional context.

At the individual level (Cooke domain 1), participants were intrinsically motivated to use research to improve practice but lacked confidence in methodology, statistical literacy and manuscript preparation. Participants revealed that their research experience was limited to mandatory undergraduate projects with little continuity after qualification. This reflects the norm of framing research as an academic requirement rather than a career-long habit, typical of many Nigerian institutions [18]. This skills gap mirrors reports from Ghana, South Africa, and even high-income countries such as Australia, where quantitative methods are often cited as barriers to research engagement [19, 20]. One-off exposures during training are insufficient to embed research as a professional norm; therefore, continuous professional development is critical for building capacity.

At the team level (domain 2), informal peer support existed but structured mentorship and team-based research activities were largely absent. In consonance with systematic reviews documenting inequities in capacity-building opportunities for allied health workers across sub-Saharan Africa, this study revealed that access to training was uneven and often self-funded, deterring engagement [2, 4]. Mentorship emerged as a strong but underdeveloped enabler of research but was frequently opportunistic and dependent on the interests of a few senior staff, leaving many without guidance and undermining confidence and productivity. Participants expressed their desire for formalised mentorship programmes and protected opportunities for team-based projects, which have been shown to boost confidence and output among allied health clinicians [21]. 

Our study showed that organisational infrastructure (domain 5) for AHP research was particularly weak; departmental research units, internal funding streams, and mechanisms for implementing research findings were absent reflecting the broader context of weak institutionalization of research in Nigeria [22]. AHPs in this study perceived research as peripheral to institutional priorities, a perception reinforced by the lack of incentives such as linking research output to career progression or departmental appraisal. Structural neglect of this nature may be more pronounced in, but certainly not unique to Nigeria: studies in Australia and the United Kingdom show that allied health research remains marginalised compared with medical research with little or no protected time and institutional recognition accorded to AHPs [20, 23]. Some participants singled out prolonged ethical approval processes as added frustration, echoing other Nigerian and African studies documenting slow, under-resourced research ethics committees [24, 25]. Our study revealed a paucity of strategic linkages and partnerships with academia, funders and professional bodies (domain 3) which ought to facilitate access to expertise, grants, and translational pathways. Participants opined that completed projects were rarely disseminated or adopted into practice or policy (domains 4 and 6) which could further erode morale and perpetuate disengagement.

Our analysis identified mechanisms by which barriers compound. For example, delayed ethical reviews stall projects, drain limited funds for pilot studies, and curb protected research time; absence of protected time lowers publication output, which in turn makes leadership view allied health research as low-value and allocate fewer resources. Breaking this cycle requires targeted, feasible interventions- small, ring-fenced institutional micro-grants to support pilot studies; formal mentorship programmes that pair early-career AHPs with experienced researchers; and streamlined local ethics review pathways or service-level agreements to expedite low-risk operational research. These interventions are supported by international evidence from allied health capacity initiatives.

Despite systemic neglect, most AHPs demonstrated resilience and an intrinsic drive to engage in research. Motivation was consistently linked to professional growth and the desire to improve patient outcomes. However, motivation alone is fragile; without competence backed by supportive structures, it is easily eroded, contributing to loss of talent and perpetuation of the brain drain documented among Nigerian health professionals [18]. Unsurprisingly, Cooke’s framework for research capacity building emphasises the importance of harnessing individual enthusiasm while embedding institutional support [10]. 

### Limitations

The paramedics cadre was underrepresented within the sample population (*n* = 4); thus, the diversity of this cadre’s experiences may not have been sufficiently captured. Since it was conducted in a single tertiary hospital, the findings may also not be representative of AHPs in other facility types or regions within Nigeria. Although the potential for social desirability bias, which is typical in group settings, may have constituted a limitation, it was mitigated by cadre-specific FGDs and assurances of confidentiality. Since recruitment relied on volunteer participation, the sample may have been biased toward staff already interested in research; however, purposive maximum-variation sampling and cadre-specific FGDs were used to mitigate this. Cadre-specific FGDs facilitated open dialogue; still, group dynamics may have suppressed some dissenting views. We triangulated FGDs with KIIs and undertook member-checking to reduce this risk.

Ethics review timelines created logistical delays during project initiation. Since we were working with timelines established by our funders, this may have negatively impacted the need for prolonged engagement with participants, a critical factor in building trust and ensuring credibility during qualitative research. However, the fact that the authors are equally staff at the study site and the use of method triangulation is likely to have made up for any lapses in credibility brought on by limited exposure.

### Implications for policy and practice

The findings from this study will be useful for strengthening allied health research capacity at the UBTH, the benefits of which are manifold for AHPs. These benefits include enhanced attitudes towards research, an increased uptake of research evidence into practice, the development of critical thinking skills and a culture of evidence-based practice as well as greater job satisfaction [16]. Strengthening allied health research capacity also has direct and measurable benefits for patient care. When AHPs lead or actively participate in research, they generate contextually relevant evidence such as improved diagnostic algorithms in laboratories, pharmacy-led medication safety audits and locally-adapted rehabilitation protocols, that can reduce length of stay, prevent adverse events, and enhance functional outcomes [2]. Institutional investments that enable small, actionable projects are likely to yield rapid, practice-relevant gains while building research literacy.

To attain these professional and patient care benefits, several reforms are required at institutional and policy levels. First, institutionalising protected research time is essential (for example, one half-day per fortnight) and this should be linked to measurable outputs. Evidence from high-income countries consistently demonstrates that dedicated time is among the strongest predictors of research output [26]. Second, structured mentorship programmes should be prioritised. Globally, mentorship is highlighted as a determinant of research career trajectories, but in Nigeria such systems remain ad hoc [16]. Third, establishing modest internal micro-grants schemes with transparent application and rapid turnaround can catalyse participation. This has been proven to significantly increase research initiation and completion rates in other African settings [27]. Revising appraisal criteria to explicitly value allied health scholarly activity, creating departmental research cells or hubs that host regular protected writing and methods sessions and streamlining ethical review processes are other ways of prioritizing and building research capacity. These reforms can serve as realistic entry points for long-term institutional change.

At the policy level, Nigeria’s national research agenda must explicitly integrate allied health cadres. Historically, research policy in Nigeria has disproportionately privileged physicians, leaving allied health workers marginalized [22]. AHPs should be included within research funding priorities, clearer career pathways for clinician-researchers should be defined, and targeted support from bodies such as the Tertiary Education Trust Fund (TETFund), professional councils, or international donors encouraged. Aligning national strategies with the needs of pharmacists, radiographers, physiotherapists, nurses, laboratory scientists, and paramedics is crucial for achieving equitable and evidence-informed healthcare. Engagement of allied health councils and professional associations will be critical in shaping reforms that are contextually appropriate and cadre-inclusive.

### Future directions

This study serves as a primer to further explore AHP research in Nigeria and other LMICs. Future research to inform policy and practice is certainly required and can take one of several directions to enrich the body of knowledge. Multisite comparative studies across Nigerian tertiary hospitals are necessary to validate or refute the findings at UBTH and quantify cadre-level differences using mixed-methods designs that combine survey measures of capacity with qualitative exploration of context. Implementation trials of short-term interventions like micro-grants, protected time and formal mentorships can be executed to assess the long-term effects on AHP research capacity. These can be undertaken using cluster randomised or stepped-wedge designs measuring outputs (project initiation, completion, publications), intermediate process indicators (attendance at mentorship sessions), and downstream patient-centred outcomes where feasible. Curriculum and CPD evaluations assessing the impact of integrated research modules (practical statistics, protocol development, writing for publication) within undergraduate and postgraduate allied health curricula can also be conducted using pre–post designs with objective competency assessments. Longitudinal cohort studies of allied health early-career clinicians to measure career trajectories, retention, and productivity following targeted capacity-building interventions should be explored by regional bodies, such as African Academy of Sciences, and global north funders, such as Wellcome Trust and NIH Fogarty, all of which have programmes actively supporting research capacity strengthening in LMICs.

## Conclusions

This study contributes an LMIC narrative to the global debate on research capacity among AHPs which has hitherto been concentrated in the global North. Our study illustrates the paradox of motivated yet constrained allied health professionals in one of Nigeria’s largest tertiary hospitals, offering detailed insights into how individual and systemic barriers shape research participation. The findings highlight much needed institutional and policy reforms-protected research time, structured mentorship, internal funding, streamlined ethics processes, and integration of research into appraisal systems- as critical enablers. Beyond UBTH, these lessons are nationally relevant and globally resonant, reinforcing that allied health research capacity building is central to health system strengthening, professional development, and the pursuit of evidence-based care.

## Supplementary Information

Below is the link to the electronic supplementary material.

Supplementary Material 1: Focus Group Discussion (FGD) Guide. Full text of the discussion guide developed for this study, outlining the introduction, ground rules, and detailed questions used to explore allied healthcare workers’ experiences, barriers, and enablers of research engagement.

Supplementary Material 2: Key Informant Interview (KII) Guide. Full text of the semi-structured interview guide developed for this study, including background questions and thematic prompts used to elicit institutional perspectives on research capacity and support for allied healthcare workers.

Supplementary Material 3: Coding theme tree.

## Data Availability

The qualitative datasets generated and analysed during the study (FGD and KII transcripts) are available from the corresponding author upon reasonable request and subject to ethical and institutional data-sharing agreements.

## Abbreviations

AHP: Allied health professional
FGD: Focus group discussion
KII: Key informant interview
LMIC: Low and middle-income countries
UBTH: University of Benin Teaching Hospital
WHO: World Health Organization
