# Supplementary material for: Exploring allied health research capacity in Nigeria: a qualitative study of enablers and barriers
Source: BMC Health Serv Res. 2025 Dec 23;26:129. doi: 10.1186/s12913-025-13942-9 (PMC12838510; doi:10.1186/s12913-025-13942-9)
Supplement: Supplementary file 1 — Supplementary Material 1: Focus Group Discussion (FGD) Guide. Full text of the discussion guide developed for this study, outlining the introduction, ground rules, and detailed questions used to explore allied healthcare workers’ experiences, barriers, and enablers of research engagement. [file 12913_2025_13942_MOESM1_ESM.docx]

**PROJECT TITLE: Capacity for healthcare research among allied healthcare workers in a Nigerian tertiary hospital**

**Focus Group Discussion Guide**

**Introduction**

- **Welcome:**
  - Introduce yourself and explain your role as the facilitator.
  - Clarify that the discussion is part of a study on enhancing research capacity and that every perspective is valuable.
  - Remind participants that their responses will be confidential and that the session is being audio-recorded (with their consent) for transcription and analysis.
- **Purpose & Ground Rules:**
  - Explain the overall goal is to understand both personal and institutional factors that influence research engagement at UBTH.
  - Emphasize open, respectful, and honest dialogue.
  - Ask participants to speak individually and avoid interruptions.

**Background questions:**

1. Could you please introduce yourself by stating your role, and the department you work in at UBTH?
2. How long have you been working at UBTH, and can you share a bit about your educational and professional background?

**Exposure to Research:**

1. Can you describe any experiences you have had with research, such as participating in clinical audits, writing research protocols, or any research projects you’ve been involved in?”

**Self-Assessment of Research Skills:**

1. How would you evaluate your own research skills, for example in areas such as literature search, writing research protocols, data collection, and analysis?
2. Please elaborate on which areas you feel most confident about and which areas you find challenging.

**Application of Research Skills:**

1. Could you share a specific example when you applied your research skills to solve a problem or improve a process in your work?

**Training and Professional Development:**

1. What kind of research training or professional development have you received so far, and how effective has it been in building your research skills?

**Motivators for Engaging in Research:**

1. What personally motivates you to participate in research? For example, do career advancement, intellectual curiosity, or the desire to improve patient care drive you?

**Influence of Professional Goals:**

1. How do your professional goals, such as gaining credibility or advancing your career, influence your decision to engage in research activities?

**Resources and Support for Research:**

1. Do you feel that you have access to adequate resources (such as equipment, funding, or software) to support your research efforts? Why or why not?

**Barriers to Personal Research Engagement:**

1. What are the biggest challenges or barriers you face when trying to engage in research activities? (For instance, do time constraints, resource limitations, or other work responsibilities play a role?)

**Departmental Support:**

1. In your experience, how supportive is your department when it comes to encouraging or facilitating research? Can you provide any specific examples or experiences?
2. How does the support or lack thereof from your department affect your motivation or ability to engage in research activities?

**Experiences with Institutional Support:**

1. How would you describe the overall environment at UBTH regarding research? Are there any hospital-level initiatives, policies, or practices that have helped or hindered your research involvement?

**Funding and Administrative Support:**

1. What has been your experience with obtaining research funding or administrative support at UBTH? Please share any specific challenges or successes you’ve encountered.”

**Use of Digital Platforms for Research:**

1. Do you utilize online platforms like ResearchGate, Google Scholar, or Academic.edu to support your research activities? How have these platforms influenced your research practice?

**Impact of Research on Professional Development:**

1. In what ways do you think engaging in research has impacted your professional development or the quality of care you provide to patients?

**Areas for Future Research:**

1. If you had the opportunity to conduct a research project on any topic of your choice, what area would you focus on and why?

**Recommendations for Improvement:**

1. What recommendations would you make to UBTH to enhance the research capacity and support available to allied healthcare workers?

**Additional Thoughts:**

1. Is there anything else you would like to add about your experiences with research at UBTH or suggestions on how the environment could be improved to better support research activities?

**Closing the Session**

- **Wrap-Up:**
  - Summarize the key points discussed during the session.
  - Invite any final comments or reflections from participants.
  - Thank the participants for their time and valuable insights
