# Supplementary material for: Exploring allied health research capacity in Nigeria: a qualitative study of enablers and barriers
Source: BMC Health Serv Res. 2025 Dec 23;26:129. doi: 10.1186/s12913-025-13942-9 (PMC12838510; doi:10.1186/s12913-025-13942-9)
Supplement: Supplementary file 2 — Supplementary Material 2: Key Informant Interview (KII) Guide. Full text of the semi-structured interview guide developed for this study, including background questions and thematic prompts used to elicit institutional perspectives on research capacity and support for allied healthcare workers. [file 12913_2025_13942_MOESM2_ESM.docx]

**Project Title: Capacity for Healthcare Research among allied healthcare workers in a Nigerian tertiary hospital**

**Key Informant Interview (KII) Guide**

**Introduction:**

Thank you for participating in this study. The objective is to explore perceptions about research engagement among non-medical healthcare workers. Your responses will remain confidential.

**Background Questions:**

1. Can you describe your role at UBTH and how long you have been in this position?
2. Have you been involved in any research projects in your career?

**Research Awareness and Knowledge:**

3. How would you describe the level of research awareness among non-medical healthcare workers at UBTH?

4. What are your thoughts on the importance of research for non-medical staff?

**Barriers to Research Engagement:**

5. What are the major barriers preventing non-medical healthcare workers from participating in research?

6. Are there organizational policies that hinder or facilitate research involvement?

**Facilitators and Recommendations:**

7. What institutional support do you think would encourage more research participation?

8. What capacity-building strategies should UBTH implement to enhance research engagement?

**Conclusion:**

9. Is there anything else you would like to add on this subject?
