# Supplementary material for: Exploring allied health research capacity in Nigeria: a qualitative study of enablers and barriers
Source: BMC Health Serv Res. 2025 Dec 23;26:129. doi: 10.1186/s12913-025-13942-9 (PMC12838510; doi:10.1186/s12913-025-13942-9)
Supplement: Supplementary file 3 — Supplementary Material 3: Coding theme tree. [file 12913_2025_13942_MOESM3_ESM.docx]

**Additional File 3: Coding Theme Tree Table**

| **Main Theme** | **Subtheme(s)** | **Sample Quotations** |
| --- | --- | --- |
| **1. Individual Research Capacity and Competence** | • Limited formal training • Self-directed learning • Variable confidence in research methods | “We were never really taught how to design or analyse research properly — most of what I know, I picked up on my own through online courses.” *(FGD 2, Physiotherapist)*  “I feel confident reading articles, but not in writing one myself or analysing data.” *(FGD 3, Nurse)* |
| **2. Institutional Support and Research Infrastructure** | • Lack of dedicated funding streams • Inadequate mentorship structures • Bureaucratic bottlenecks in ethics and approvals | “Even when you have a good research idea, there is no internal funding to push it forward.” *(KII 1, Departmental Head)*  “Sometimes approvals take months — by the time you get feedback, the motivation has died.” *(FGD 4, Laboratory Scientist)* |
| **3. Cultural and Organisational Barriers** | • Perceived undervaluation of allied health research • Clinical workload pressures • Limited recognition of research outputs in appraisal | “Here, research by allied health staff is not really recognised or rewarded, so most people just focus on patient care.” *(FGD 5, Radiographer)*  “We are always short-staffed; there’s hardly time left after clinical duties.” *(FGD 1, Nurse)* |
| **4. Motivators and Enablers for Research Engagement** | • Institutional leadership encouragement • Peer collaboration and networks • Desire for professional growth and visibility | “When management acknowledges and supports our efforts, it really motivates us to do more.” *(KII 3, Departmental Head)*  “Collaborating with colleagues from other disciplines helps us see research as a team effort.” *(FGD 6, Paramedic)* |
| **5. Strategic Recommendations and Future Directions** | • Need for structured capacity-building programmes • Policy alignment with research incentives • Integration of research into routine practice | “A structured research mentorship and short-term training would go a long way in building our confidence.” *(KII 4, Unit Head)*  “Research should be seen as part of our job description, not an extra burden.” *(FGD 2, Pharmacist)* |
